# Understanding Unrest: Conspiracy Belief and Violent Radicalization Patterns in Young People During the COVID-19 Pandemic in the Netherlands

**DOI:** 10.1007/s10610-025-09634-z

**Published:** 2025-08-21

**Authors:** Hanne M. Duindam, Jessica J. Asscher, Friedrich Lösel

**Affiliations:** 1 Faculty of Social Sciences, Clinical Child, Family and Education Studies, Utrecht University, Utrecht, Netherlands; 2 Darwin College, University of Cambridge, Cambridge, England; 3 Institute of Criminology, University of Cambridge, Cambridge, England; 4 Institute of Psychology, Friedrich-Alexander-University Erlangen-Nuremberg, Erlangen, Germany

**Keywords:** Conspiracy belief, COVID-19, Violent radicalization, Person-centered analysis, Risk factors

## Abstract

Conspiracy theories threaten Western societies’ cohesion and democracy. Yet, little is known about who might be vulnerable to conspiracy-motivated violent radicalization, which is essential for effective prevention. The first aim of this study was to identify profiles of cognitive and behavioral violent radicalization tendencies and COVID-19 conspiracy exposure and belief among a sample of Dutch youth (*N* = 593, 16 to 26-years-old) drawn during the COVID-19 pandemic. The second aim was to describe the identified profiles based on risk factor presence. Latent-profile analyses identified four profiles. Most individuals belonged to the general population profile (64%) characterized by low levels of cognitive and behavioral violent radicalization, as well as low levels of COVID-19 conspiracy exposure and belief. The experimenters (20%), violent-risk (8%), and conspiracist (7%) profiles displayed higher levels of these constructs. Results revealed that micro- and meso-level risk factors were most pronounced in the violent-risk profile, which exhibited the highest levels of behavioral violent radicalization. For people in the conspiracist profile with higher levels of COVID-19 conspiracy belief, micro-level risk factors, specifically police illegitimacy and perceived personal discrimination were important, demonstrating the importance of fairness and justice during political decision-making. Overall, integrating evidence-based strategies for anti-crime interventions into anti-radicalization efforts may help curb violently radical behavior (Jugl et al., *Monatsschrift Fur Kriminologie Und Strafrechtsreform*, 1–16. 2021a).

**Supplementary Information:**

The online version contains supplementary material available at 10.1007/s10610-025-09634-z.

## Introduction

Conspiracy theories (CTs) are considered a threat to cohesion and democracy in current Western societies (AIVD, 2023). CTs have influenced people’s public health responses to the COVID-19 pandemic, defined the course of election campaigns (e.g., Brexit), and undermined effective responses to climate problems (Biddlestone et al., 2022; Bierwiaczonek et al., 2022). Concerns about conspiracy belief fostering violent radicalization have been raised. Conspiracy belief can be defined as the belief in conspiracy theories (CTs), which are narratives suggesting that actor(s) secretly coordinate plans to achieve malevolent goals (van Prooijen, 2020). Most people who believe in CTs will never become violent. However, there are several high-profile instances whereby conspiracy belief has been linked to violent radicalization in society, such as the recent storming of the U.S. capitol (Basit, 2021). Most previous research on the topic has adopted a variable centered approach, examining conspiracy belief as a risk factor for violent radicalization. However, this approach overlooks how conspiracy belief and violent radicalization may intersect. Moreover, it does not capture diversity in youth’s belief and behavioral tendencies. To address this gap, the present study has adopted a person-centered approach, which allows for the identification of specific profiles based on individual variation on a combined set of variables (Bámaca-Colbert & Gayles, 2010; Howard & Hoffman, 2018). The first study aim is to investigate profiles of cognitive and behavioral violent radicalization tendencies and COVID-19 conspiracy theory belief and exposure among a sample of young Dutch people during the COVID-19 pandemic. To better understand the background of the youth in these different subgroups, the relevance of demographic factors is assessed. The second study aim is to test to what extent risk factor presence differed between more- and less- at-risk subgroups.

### Violent Radicalization in Young People

Violent radicalization is considered a process by which a person adopts beliefs that justify the use of violence for social and political change (Doosje et al., 2016; Lösel et al., 2018). This process is context-dependent, and attitudes and behavior are considered violently radical when they significantly deviate from society’s dominant political and legal norms and seek at least partial system abolishment through violence (Beelmann, 2020).

Violent radicalization can be cognitive including attitudes, such as sympathizing with the use of violence or the willingness to commit violence (cognitive violent radicalization). For a select few (< 1%), violent radicalization is behavioral when violent acts are committed for ideological beliefs (Wolfowicz et al., 2021). Most people who support violence for societal change (cognitive violent radicalization), will not act violently (Moskalenko & McCauley, 2021). However, those who do behaviorally radicalize, most likely will have been cognitively radicalized first (Lösel et al., 2018; Moghaddam, 2005). The majority of research has focused on cognitive violent radicalization (Wolfowicz et al., 2021), whereas radical violence can arguably have more detrimental consequences for society. Therefore, the current study encompasses both cognitive and behavioral aspects of violent radicalization. For behavioral violent radicalization, actions targeting both people and property are considered. In the context of violent radicalization, property damage tends to be a more common form overall and can sometimes serve as a precursor to violence against people. Both forms share some risk factors while also exhibiting distinct ones (e.g., Jugl et al., 2021a; Pauwels & De Waele, 2014).

In this study, the focus is on young people, as violent radicalization is most likely to emerge in adolescence through middle adulthood (Beelmann, 2020). Young people appear more prone to extremist milieus due to their developing identities, search for recognition and belonging, and increased tendency for provocation (Schröder et al., 2022). Their immersion online, coupled with a lack of digital skills (Breakstone et al., 2021; Loos et al., 2018; Prievara et al., 2019), which may also contribute to a heightened proneness to CTs.

### Conspiracy Belief and Violent Radicalization

Government officials have expressed concern about a potential new form of terrorism that is based on conspiracy belief (e.g., Pantucci, 2020). New forms of violent radicalization that are not neatly linked to specific religious or political ideologies, but based on conspiracy belief and ‘salad bar ideologies‘ (i.e., a mix of loosely connected ideas taken from different ideologies) seem to have become more apparent (Basit, 2021; Gartenstein-Ross et al., 2023). Examples include the storming of the US capitol related to QAnon, 5G towers being lit on fire motivated by belief in COVID-19 CTs, and people avoiding the payment of taxes related to belief in the sovereign citizen CT. Fortunately, most conspiracy believers do not become violent (Moskalenko & McCauley, 2021). Yet, belief in conspiracy theories is widespread, and the question has been raised when and for whom they might exacerbate the risk for violent radicalization (Kruglanski et al., 2022; Rottweiler & Gill, 2020).

### Theoretical Framework

An essential component of the violent radicalization process is the adoption of narratives that (1) frame grievances or (perceived) injustices and (2) justify the use of violent or illegitimate methods as a counter-response (e.g., A Social-Developmental Model of Radicalization, Beelmann, 2020; the 3 N Model of Radicalizaiton, Kruglanski et al., 2022). Within that context, belief in CTs are conceptualized to play an important role (Bartlett & Miller, 2010; Basit, 2021; Kruglanski et al., 2022). Some argue that a conspiracy mindset can function as a generalized political attitude in itself (Imhoff & Bruder, 2014), characterized by a general distrust of power, distinct from other recognized political attitudes (e.g., right-wing authoritarianism). Conspiracy theories tend to provide an account of the world that is simplified and intuitive. They can make complex events and one’s role within them more understandable and predictable (Vegetti & Littvay, 2022). This perspective is often provided by zooming into a specific threat (e.g., economic crisis) and identifying an out-group responsible for the apparent threat (e.g., the elite). Such a unifying narrative about an evil, responsible enemy can promote us-versus-them thinking and deepen group divisions, potentially leading to the dehumanization of the ‘other’– processes often viewed as integral to violent radicalization (Bartlett & Miller, 2010; Basit, 2021). Thereby, belief in CTs is deeply woven into the process of violent radicalization: CTs provide the narratives that help channel feelings of resentment onto specific targets. These narratives may be fundamental to the process of violent radicalization by guiding individuals along the path toward increasingly extreme and sometimes violent attitudes and actions (Bartlett & Miller, 2010; Vegetti & Littvay, 2022). The 3 N Model of Radicalization highlights how a social network that endorses violence can contribute to the risk of conspiracy belief escalating into violent behavior (Kruglanski et al., 2022).

### Previous Research

***CT exposure and violent radicalization.*** An increasing number of studies have examined the relation between exposure to CTs and violent radicalization. First, there are significant concerns about the effects of exposure to CTs on attitudes and behaviors. This is because a large amount of misinformation, of which CTs are a part, can be found online (Lee et al., 2020) and because repeated exposure to misinformation increases its perceived accuracy (Pennycook et al., 2018). Mere exposure to CTs has been found to influence behavior, regardless whether people believe them or not (e.g., Douglas & Sutton, 2008). CT exposure can by itself arouse strong emotions in people (e.g., anxiety, anger), which could create the psychological motivation for (support of) a violent response (Oberschall, 2012; Roberts-Ingleson & McCann, 2023). There is some evidence in support of this hypothesis, which suggests that exposure to more radical forms of misinformation content online can increase the risk for radical attitudes and behavior (Wolfowicz et al., 2022). However, there is a lack of empirical research on the relation between misinformation exposure – or CT exposure in particular – and radicalization (Roberts-Ingleson & McCann, 2023).

***CT belief and violent radicalization***. The relation between belief in CTs and violent radicalization has been more frequently examined. In general, an association has been found between the endorsement of (COVID-19) CTs and support for violent radicalization (Levinsson et al., 2022), acceptance of violence as a tolerable way to express disagreement with the government (Uscinski & Parent, 2014), and having greater violent radicalization intentions overall (Jolley & Paterson, 2020; Rottweiler & Gill, 2020). Even running a red traffic light may be related to such attitudes (Jolley et al., 2019). Overall, the relation between conspiracy belief and violent radicalization appears quite small. For some people conspiracy belief appears to play a more important role. A recent analysis of the manifesto’s of those engaged in acts of self-sacrificial violent radicalization revealed that the majority of offenders referenced conspiracy theories (Ebner et al., 2022). Some evidence also suggests that CT belief may be differently associated with violent radicalization, depending on the nature of the CT (Levinsson et al., 2022). However, most previous research has examined conspiracy beliefs as a composite, combining belief in different CTs or looking at a general tendency to believe in CTs (Imhoff et al., 2022).

### Risk-Factor Approach

Studies agree that violent radicalization and conspiracy belief can both result from the accumulation and interaction of risk factors across different levels (Hornsey et al., 2022; Miconi et al., 2021b).

According to the socioecological framework, risk factors can be present at the micro (individual), meso (group), and macro-level (societal) level. The current study focused predominantly on micro-level risk factors such as an individual’s characteristics and experiences (e.g., aggression, perceived procedural injustice), but also included a group level risk – that is peer delinquency – as a meso-level risk factor. Even though no direct macro-level (societal) factors were measured in the present study, the societal situation at the time of data-collection in the Netherlands is relevant and will be discussed. Below follows a discussion of unique and shared risk factors for conspiracy belief and violent radicalization.

#### Micro-Level Risk Factors

Both conspiracy belief and violent radicalization are seated in a deep distrust of governmental institutions, with grievances and injustice playing a central role (Basit, 2021; Murphy et al., 2022). *Perceived procedural injustice* can be seen as a component of this distrust and has been identified as a risk factor for both conspiracy belief and cognitive and behavioral violent radicalization (Emmelkamp et al., 2020; Pilch et al., 2023; van Prooijen, 2018). The perception of unjust procedures can result in ‘anomie,’ or the breakdown of shared norms and values that support institutional functioning and are essential for people’s adherence to law and order (i.e., Procedural Justice Theory; Pauwels & De Waele, 2014), increasing the appeal of conspirational narratives and making violent radicalization more likely. For similar reasons, p*erceived police illegitimacy* has been identified as a risk factor for violent radicalization. A recent meta-analysis with youth found perceived illegitimacy of authorities to constitute as a small risk for cognitive and behavioral forms of (violent) radicalization (Emmelkamp et al., 2020), including violence towards people and property (Pauwels & De Waele, 2014). For conspiracy belief, political illegitimacy is also expected to play a role, as many studies have demonstrated a link with distrust in institutional representatives more generally (for a recent review see Pilch et al., 2023).

*Perceived personal discrimination* can foster both violent radicalization and conspiracy belief. The experience of discrimination causes strain, creating distress which can drive people to radical ideologies and thereby increase the risk for violence as a form of perceived retribution (i.e., General Strain Theory, Agnew, 2010, 2011). Accordingly, perceived personal discrimination has been identified for cognitive violent radicalization in adults and youth (Emmelkamp et al., 2020; Wolfowicz et al., 2021). For the latter group, personal discrimination was also weakly associated with behavioral (violent) radicalization (Emmelkamp et al., 2020), although one study found its role quite minor in both violence and property towards people (Pauwels & De Waele, 2014). Generally, a heightened level of conspiracy belief has also been found in communities with experience of discrimination and mistreatment (Bilewicz, 2022; van Prooijen & Douglas, 2017).

*Aggressiveness* and *impulsiveness* have been positively associated with cognitive and behavioral violent radicalization (Simi et al., 2016; Wolfowicz et al., 2021) possibly because they are indicative of underlying antisocial and rule-breaking behavioral tendencies (Nivette et al., 2017). One study found how impulsiveness was a risk factor for political violence towards people but not towards property (Pauwels & De Waele, 2014). For conspiracy belief, a direct association with aggression is not expected, although a relation between impulsiveness and COVID-19 conspiracy belief has been found previously (Alper et al., 2021).

*Changes in daily life*, meaning negatives changes as a result of the COVID-19 pandemic (e.g., being out of work), could also contribute to a higher cognitive violent radicalization risk through strain (Jolliff et al., 2021). In general, historical trends demonstrate that during times of crises, with aversive effects on people’s daily life, conspiracy belief increases. The fear, anxiety, and uncertainty that people might experience during times of crises activate sense-making mechanisms that heighten the tendency to perceive conspiracies in social contexts (van Prooijen & Douglas, 2017).

#### Meso-Level Risk Factors

Recent review studies demonstrate how *peer delinquency* is a significant risk factor for crime generally and for cognitive and behavioral violent radicalization specifically (Assink et al., 2015a; Emmelkamp et al., 2020; Wolfowicz et al., 2021), with nearly all theoretical models emphasizing that people, as social beings, are influenced by the behavior of others in their environment (i.e., Social Learning Theory; Akers, 2009; Pritchett & Moeller, 2022). No relation is expected between peer delinquency and conspiracy belief.

***Macro-level risk factors*** were not assessed in the present study. Still, it is important to consider the Dutch socioenvironmental context during the COVID-19 pandemic, and specifically during data-collection between February and July 2022. During the COVID-19 pandemic the Netherlands had adopted a self-coined “intelligent lockdown” approach, with an initial focus on the protection of healthcare system, economy, and vulnerable people (soon the focus narrowed to solely maintaining intensive care capacity). Whereas its initial lockdown was relatively light (i.e., initially schools and day-care centers stayed open), more drastic measures were adopted as the pandemic continued, making the Netherlands similar to other European countries (Van Dullemen & De Bruijn, 2021).

At the time of data-collection in the first half of 2022, the Netherlands had entered one of its latter phases of the COVID-19 pandemic. Restrictions were being phased out and schools and universities had just re-opened. The country was still partially in lockdown, for example, shops were open until 17:00 only. During the study period society was slowly reopening and in April 2022 Dutch citizens did not have to quarantine anymore after a positive COVID-19 test. In May 2022 it was no longer obligatory to wear masks at the airport. A citizen’s poll at the time demonstrates that while concerns about the COVID-19 pandemic were reducing, concerns about the economy, political unrest, the war in Ukraine, and polarization were increasing. Respondents increasingly experienced frustration about a small group of people with, in their eyes, extreme opinions. Trust in politics continued on its downward trend in the beginning of 2022 with only around 50% expressing confidence in the government (Miltenburg et al., 2022).

#### Sociodemographic Factors

Finally, to understand heterogeneity in conspiracy belief and violent radicalization, research has also considered specific sociodemographic factors, such as age, gender, cultural background, and educational achievement. For example, younger people have been found more vulnerable to conspiracy belief and violent radicalization (Bordeleau & Stockemer, 2024; Carlsson et al., 2020). Male youth, especially those with a criminogenic background, have been found to be more at risk for violent radicalization (Emmelkamp et al., 2020), whereas the associations found between gender and conspiracy belief has been more mixed (Enders et al., 2024). With regards to cultural background and a potential relation with violent radicalization, second generation immigrants have been found to be more at-risk (Miconi et al., 2021), which might be driven by group-based experiences of deprivation and discrimination (Obaidi et al., 2019). Finally, while lower educational level has mostly been linked with higher conspiracy belief (van Prooijen, 2017) and violent radicalization tendencies (Emmelkamp et al., 2020), sometimes opposite results are found (Roscigno, 2024).

### The Current Study

By adopting a person-centered approach, the first objective of the current study is to examine to what extent meaningful profiles can be identified based on cognitive and behavioral violent radicalization and COVID-19 conspiracy exposure and belief in a sample of Dutch youth (16-to-26-years-old). Person-centered approaches can complement the more common variable-centered methods to study individual profiles or prototypes of configurations (e.g., Bergman & Lundh, 2015; von Eye & Wiedermann, 2015). Person-centered analyses give more insight into the heterogeneity of characteristics in a population, also revealing the relation between variables in each subgroup, thereby providing more context for interpretation. To better understand the background of the youth in the different subgroups, the relevance of demographic factors is also assessed.

The second objective of this study is to test differences between profiles in terms of micro- and meso-level risk factor presence. Understanding risk factor level presence of different subgroups can give insight into more clearly defined target groups for prevention and intervention efforts, which is crucial to increase the effectiveness of these programs (Howard & Hoffman, 2018). Although we have hypotheses about the risk factor variables (see above), no specific hypotheses are formed about what profiles would be identified due to the exploratory nature.

## Methods

**Sample**. A total of 593 participants, aged 16 to 26, participated in the current study by completing a digital survey. Participants were recruited in educational settings, online, and at events (see procedures for detailed description). There were no inclusion criteria other than participants’ age (i.e., 16-to-26-years-old). Participants (*N* = 593) were on average 21.2 years-old (*sd* = 3.00). The majority were female (72.7%, *n* = 431), whereas a smaller subgroup identified as male (25.6%, *n* = 152) and a minority identified as other (1,7%, *n =* 10). Participants were recruited in educational settings across the Netherlands, online, and during specific events (e.g., demonstrations against government restrictions to curtail the COVID-19 spread). Most participants indicated that they found the research on social media (*n* = 240, 40.5%), followed by educational settings (*n* = 160, 27.0%). Others reportedly received the link to the survey because it was sent to them by friends or family (*n* = 85, 14.3%), shared on a group app they were part of (*n* = 26, 4.4%), provided to them at work (*n* = 14, 2%), or received in another manner (*n* = 68, 11.5%. e.g., through a QR code at an anti-government restriction demonstration). Most participants were enrolled in education, such as secondary school (*n* = 95, 16.0%) or a form of tertiary education (*n* = 330, 55.6%). Others worked full- (*n* = 121, 20.4%) or parttime (*n* = 34, 5.7%). A smaller portion of participants indicated that they were unemployed (*n* = 13, 2,2%). Most participants were born in the Netherlands and had parents who were born in the Netherlands (*n* = 546, 92.1%), a small minority was born elsewhere (*n* = 28, 4.7%) or had one or two parents who were born elsewhere (*n* = 19, 3.2%).

In total, 715 participants had started the digital questionnaire and provided at least information on demographics, their COVID-19 CT belief, and one risk factor variable. 6 participants were removed because they were either under 16-years-old, or had extreme and unrealistic age values (−0.42, 122), resulting in 709 participants. As the focus of this study was on 16 to 26-year-olds, an additional 116 participants were removed who were older than 26-years.

**Procedure.** Ethical approval for this study was obtained prior to the start of data collection from the Ethical Committee of the Faculty of Social and Behavioral Sciences of Utrecht University (No. 21–0498). This study was preregistered (https://osf.io/e4652) and the informed consent form and questionnaire were presented in Qualtrics. Prior to study participation, participants were presented with a digital informed consent form, and all participants gave their approval before continuing to the questionnaire (i.e., in the Netherlands, participants can give informed consent independently from parents starting at age 16 and older). The informed consent form contained all information about the study topic, procedure, participation, confidentiality, voluntary nature of participation, and contact information in case of questions or complaints. Participants were informed that the questions would address key societal topics, including their perception of the COVID-19 virus, related restrictions, government and police actions, experiences with discrimination, and their views on and experiences with violence. They were also informed beforehand that they would be asked about sensitive topics, such as whether they had been convicted.

The digital questionnaire was offered in educational settings and online. Several different types of educational settings were visited, such as high schools and institutions offering tertiary educational programs. Usually, the questionnaire was completed during a class, however, sometimes the link to the questionnaire was distributed through the schools electronic learning environment. Research assistants (i.e., trained Master students who helped with data collection for their thesis) at Utrecht University assisted with the administration of the questionnaire in the classroom. They introduced the research and were available to answer any questions from participants. To reach people with potentially higher levels of COVID-19 CT exposure and belief, research assistants also distributed the links on online platforms and in messaging groups and Facebook/Instagram pages that posted contents related to conspiratorial thinking. Moreover, some anti-COVID-19 restriction demonstrations and festivals were visited to recruit participants.

### Materials

Unless stated otherwise, instruments used a 5-point scale ranging from 1 (*completely disagree*) to 5 (*completely agree*). Mean scores were calculated, unless stated otherwise, with higher scores indicating a higher level of the measured variable. Omega’s, means, standard deviations, and correlations of all constructs are reported in Table 1.

***Profile variables.*** *COVID-19 conspiracy theory belief (CT beliefs)* was assessed by asking participants to rate their agreement with seven conspiracy beliefs about COVID-19 on a 5-point scale ranging from 1 (*disagree*) to 5 (*fully agree*). These specific COVID-19 conspiracy beliefs were chosen because they were assessed in previous (Dutch) research during the pandemic (Van Bavel et al., 2022; van Prooijen et al., 2021; Prooijen et al., 2022a, b). Examples include: “The COVID-19 virus is a conspiracy to take away citizens’ rights for good and establish an authoritarian government” and the “COVID-19 virus outbreak is related to the construction of the 5G Internet network.”

*Exposure to COVID-19 conspiracy theories (CT exposure)* was assessed by asking participants how often they were exposed to the seven COVID-19 conspiracy theories online or, for example, on social messaging or via social media (Lee et al., 2020) on a 4-point scale ranging from 1 (*never*) to 4 (*often*).

*Cognitive violent radicalization*, in the form of sympathy for radicalization, was assessed by using a Dutch translation of Sympathies for Violent Radicalisation and Terrorism scale (Bhui et al., 2014). Participants were asked to rate their support for/condemnation of eight different actions conducted as part of political protests (e.g., commit minor crime, use violence, use bombs to fight injustice).

*Behavioral violent radicalization* in the form of violence towards *people* was assessed by asking participants if they had ever “fought with someone,” “thrown stones at the police during a demonstration,” “threatened anyone on the internet,” “threatened someone in the street,” and “hit someone” because of their political or religious beliefs (Pauwels & De Waele, 2014). Participants selected how often they had engaged in the violently radicalized behaviors on a 4-point scale, with 0 being *never*, 1 being *one time*, 2 being *two-to-three times*, and 3 being *more than three times*. Item responses were dichotomized, to the presence (1) or absence (0) of any form of behavioral radicalization.

*Behavioral violent radicalization* in the form of violence towards *property* was measured by asking participants if they had ever “vandalized anything in the street or at a station,” “damaged someone’s property,” and “set something on fire” because of their political or religious beliefs (Pauwels & De Waele, 2014). Participants selected how often they had engaged the radicalized vandalism on a 4-point scale, with 0 being *never*, 1 being *one time*, 2 being *two-to-three times*, and 3 being *more than three times*. Item responses were dichotomized, to the presence (1) or absence (0) of any form of behavioral radicalization.

*Demographic characteristics* included age, gender (man/boy, woman/girl, other), cultural background, and educational achievement. Cultural background was dichotomized as participants who were born in the Netherlands and who had parents who were also born in the Netherlands (‘native’), and participants who were born in another country or whose parents (at least one) were born in another country were considered (‘immigrant’). In addition, educational achievement reflected participants’ self-perception with regards to their educational performance. A dichotomous variable was created for the following two levels: perform(ed) well (sometimes) and did not perform well/perform(ed) terribly.

**Micro-level risk factors.** *Perceived procedural injustice* gave insight into the extent to which participants experienced the government as just in their treatment of citizens and was measured by five adjusted items (Jackson et al., 2012), such as “the government… takes the time to listen to people” and “… takes fair and impartial decisions.” All items were reverse coded.

*Perceived police illegitimacy* was measured by assessing participants’ obedience to and moral alignment with the police using eight items based on the European Social Survey (e.g., “I back the decision made by the police even when I disagree with them,” “Police have the same sense of right and wrong as me,” Jackson et al., 2012; Pauwels & De Waele, 2014). All items were reverse coded.

*Perceived personal discrimination* was measured using four times (Pauwels & De Waele, 2014; Van den Bos et al., 2009) including, among others: “I have the feeling of being discriminated.”

*Aggression* was measured on a scale from 1 (*never*) to 3 (*often*) using the 17 items of the Aggression subscale from the Youth Self Report Form (Achenbach & Rescorla, 2001). Example items are: “I fight often” and “I am mean to others”.

*Impulsiveness* was assessed using four items (Grasmick et al., 1993; Pauwels & De Waele, 2014), such as: “If I want something, I do it immediately” and “I lose my temper easily.”

*Changes in daily life experiences due to the pandemic* was assessed by asking participants if pandemic prevented them from working (1), going to school (2), taking part in extracurricular activities (3), and/or exercise (4) (Jolliff et al., 2021). One point was allocated for every prevented activity, and the total score was a sum of all activities that could not be carried out due to the pandemic.

**Meso-level risk factor.** *Peer delinquency* was measured using five items (Pauwels & De Waele, 2014) that asked participants about the delinquent actions of their peers (e.g., Have you friends been involved in… taking something from a shop/supermarket?,… stealing money or other goods from somebody?,…hitting someone on purpose so that the person needed care?). Participants indicated per item whether none (*0*), some (*1*), most (*2*), or all (*3)* of their friends had been involved in the delinquent actions.

### Analyses

For a subset of the 593 participants (*n* = 156, 22%), some data were missing. Missing data were handled using the full-information maximum likelihood estimator with robust standard errors (MRL), which accommodates for non-normality and non-independence (Múthen & Múthen, 2017b). Descriptive analyses (i.e., Pearson correlations, means, standard errors, omega’s) were run to better understand the data.

#### Latent Profile Analyses

To assure data quality, minimum response-speed (i.e., 1 s per item or higher; Wood et al., 2017) was checked before conducting the analyses. In addition, to check for patterns of careless responding by repeatedly selecting the same response options in the profile variables, the longest string of identical responses for each participant was identified through the long-string analysis. Such straignlining behavior is a common indicator of inattention (Curran, 2016). Participants with long-string scores exceeding three median absolute deviations above the median were excluded.

Then, a confirmatory factor analysis was conducted to determine whether each profile variable aligned with its underlying factor structure. Next, to identify profiles of cognitive and behavioral violent radicalization and COVID-19 CT exposure and belief, a latent profile analysis (LPA) was performed in Mplus 8.0 using the latent factor scores of the profile variables. First, a single-profile model was tested. Subsequently, two, three, four, and five profile-models were tested. Various statistics were used to evaluate model fit (see Table 2), including Akaike Information Criterion (AIC), Bayesian Information Criterion (BIC), and Adjusted-BIC, with smaller values indicating more optimal fit. Entropy values were also examined as they are indicative of the accuracy of class membership, with higher values indicating a more accurate classification of individuals. Overall, the best profile model was determined based on model fit statistics, entropy values, parsimony, and interpretability (Berlin et al., 2014). In addition, Lo-Mendell-Rubin Likelihood Ratio tests were conducted to test to what extent each additional profile model significantly improved model fit. After selection of the best model fit, the posterior distribution of the latent profile and model class assignment for the best-fitting model were saved. A nominal variable indicating the most likely class was generated, and the average misclassification error was calculated. For each profile, standardized latent means along with standard error estimates were requested for the cognitive and behavioral violent radicalization and COVID-19 conspiracy exposure and belief variables (STDYX, Mplus). Lastly, the final profile membership of each case was saved, and Wald tests were conducted to examine to what extent at-risk profiles differed from the no-risk profile based on CT exposure, CT belief, cognitive violent radicalization, behavioral violent radicalization (towards people and property). Differences between no-risk and at-risk profiles in the demographic variables of age, gender, cultural background, and educational achievement were also assessed, using the two-step Bolck-Croon-Hagenaar (BCH) method (Asparouhov & Muthén, 2019; Spurk et al., 2020). This method preserves the latent profile structure by incorporating the demographic factor independently as auxiliary variables into the model, ensuring that associations with profile membership are unbiased by misclassification errors.

#### Risk Factor Presence

To assess the relation between the identified profiles and risk factors, and test the difference between at-risk and no-risk profiles in terms of risk factor presence, the same two-step BCH method described above was applied (Asparouhov & Muthén, 2019; Spurk et al., 2020). Standardized values (below and above the mean) are reported per profile for each risk factor.

#### Power and Consistency of Findings

Monte Carlo simulations were run with 1000 replications to determine power levels (for an elaborate overview of procedures, see Muthén & Muthén, 2017a). Power levels were determined by examining the proportion of replications that were statistically significant. To assess the consistency of the findings, parameter and standard error bias estimators were generated. Finally, to check the precision of the model estimates, coverage rates were computed to check accuracy of confidence intervals.

#### Multiple Testing

The Benjamini-Hochberg correction (1995) was applied to address multiple testing.

## Results

### Descriptive Data

All participants passed the minimum response-speed and the long-string analysis (Curran, 2016; Wood et al., 2017). Descriptive data can be found in Table 1 for all variables. Overall, low mean values were found for COVID-19 exposure and belief and cognitive and behavioral violent radicalization. Most profile variables were at least weakly correlated, except for COVID-19 CT exposure with cognitive violent radicalization and behavioral violent radicalization (violent towards property). Most demographic characteristics and risk factors were weakly to moderately correlated with some profile variables. The strongest correlation was found between age and radical attitudes.

**Table 1:** Correlation matrix of latent profile variable factor scores, demographic characteristics, and standardized risk factors

	1	2	3	4	5	6	7	8	9	10
1. COVID-19 CT exposure	-									
2. COVID-19 CT belief	.275***	-								
3. Cogn. Rad	.057	.135***	-							
4. Beh. Rad (ppl)	.135**	.087***	.093***	-						
5. Beh. Rad (prop)	.031	.053***	.117***	.290***	-					
6. Gender	-0.022	.059	.151***	.262***	.223***	-				
7. Age	0.003	−.151	−.608***	−.326	−.416***	−.212***	-			
8. Cult back	-0.046	−.136**	−.038	−.048	−.063	−.147	.067	-		
9. Edu. ach	−.248	.091	**.150**	.131*	.233***	.554***	**.003**	−.032	-	
10. Proc. injustic	.044	.096	.074**	** −.041**	−.063*	−.167***	.005	.065	.037	-
11. Pol. illegit	.010	.323***	.255***	.165***	.120***	.016	−.192***	−.258***	.099	.355***
12. Pers. discr	.154**	.223***	.187***	.108***	.133***	.125*	−.108*	−.257***	.181	.209***
13. Aggression	.101	−.021	.187***	.194***	.166***	.053	−.286***	−.038	.227	.236***
14. Impuls	−.104*	.053	.176***	.001	.108***	.148*	−.255***	.004	.266*	.010
15. Changes life	−.001	−.036	.060*	−.027	.004	−.223*	.029	.012	.068	.165***
16. Peer delinq	.087	−.009	.186***	.231***	.268***	.233***	−.198***	−.054	.321***	.033

	11	12	13	14	15	16	*M (sd); %*^*1*^	Ω	% complete	
1. COVID-19 CT exposure							1.35 (.71)	0.89	100%	
2. COVID-19 CT belief							1.84 (.66)	0.93	100%	
3. Rad. att							1.75 (.72)	0.91	80%	
4. Viol							4%^1^	0.81	90%	
5. Van							6%^1^	0.79	90%	
6. Gender							-	**-**	100%	
7. Age							-	-	100%	
8. Cult back							-	-	100%	
9. Edu. ach							-	-	100%	
10. Proc. injustic							-	0.90	89%	
11. Pol. illegit	-						-	0.89	86%	
12. Pers. discr	.367***	-					-	0.88	83%	
13. Aggression	.289***	0.248***	**-**				-	0.80	77%	
14. Impuls	.123**	0.124***	.416***	**-**			-	0.68	81%	
15. Changes life	.140***	0.167***	.138***	.109**	**-**		-	-	73%	
16. Peer delinq	.245***	0.182***	.385***	.291***	.054	-	-	0.71	87%	

*Notes*. CT = Conspiracy Theory, Cogn. Rad. = cognitive violent radicalization, Beh. Rad: behavioral violent radicalization (% of people indicating at least one form of behavioral violent radicalization), ppl = people, prop = property, Gender (0 = woman/girl, 1 = man/boy), Cultural background (0 = ‘native’, 1 = immigrant), Educational achievement = self-reported educational achievement: “good” performance (0), “bad/terrible” performance (1), proc. = procedural, pol. = political, pers. = personal, impuls. = impulsivity, delinq. = delinquency, %^1^: percentage of sample who reported having engaged in violently radical behavior towards people and property, % complete = percentages of respondents with complete data on this variable

^*^ *p* <.05, ** *p* <.01, *** *p* <.001

### Profiles

Results of the Confirmatory Factor Analysis testing the 5-factor model^1^ demonstrated an excellent fit χ² = 542.036, *p* <.001, CFI = 0.966, TLI = 0.963, and RMSEA = 0.022 (90% CI: 0.016–0.027) with an acceptable SRMR of 0.066 (see Appendix). Table 2 shows the fit indices of the latent profile analyses for the one, two, three, four, and five profile models. The four-profile model was selected as the best model because it provided the most parsimonious, well-fitting, and conceptually meaningful solution with good model-fit criteria. This model showed a better fit than the 3-profile model, based on the three information criteria (AIC, BIC, and adjusted BIC) and results of the Lo-Mendell-Rubin Ratio test. Even though it had a marginally lower entropy value than the three-profile model, it was conceptually the most meaningful as it identified a fourth profile with high level of conspiracy belief. The 5-profile model had slightly better model test statistics than the 4-profile model, including a marginally higher entropy, but did not result in a conceptually meaningful solution as the additional profile appeared to further split one smaller profile (see Appendix).

**Table 2:** Latent Profile Analysis Indices of Model Fit

Number of classes	AICa^a^	BIC^b^	Adjusted BIC^c^	Entropy^d^	Smallest profile size	LMRT
1	5456.581	5500.433	5486.686	-	593	
2	4277.124	4347.287	4296.492	0.955	113	1132.345***
3	3660.342	3756.817	3686.974	0.951	50	597.586***
4	3266.881	3389.667	3300.775	0.944	44	385.343***
5	3017.399	3166.496	3058.556	0.954	12	248.511***

*Note*. AIC = Akaike Information Criterion, BIC = Bayesian Information Criterion, LMRT = Lo-Mendell-Rubin ad-hoc adjusted likelihood ratio test

^***^ p <.001

^a^ Lower values indicate a better fit (McLachlan & Peel, 2000)

^b^ Lower values indicate a better fit (McLachlan & Peel, 2000)

^c^ Lower values indicate a better fit (McLachlan & Peel, 2000)

^d^ Represents the accuracy of class membership, with higher values inidicating better categorization of persons in subgroups (Berlin et al., 2014)

**Fig. 1:**
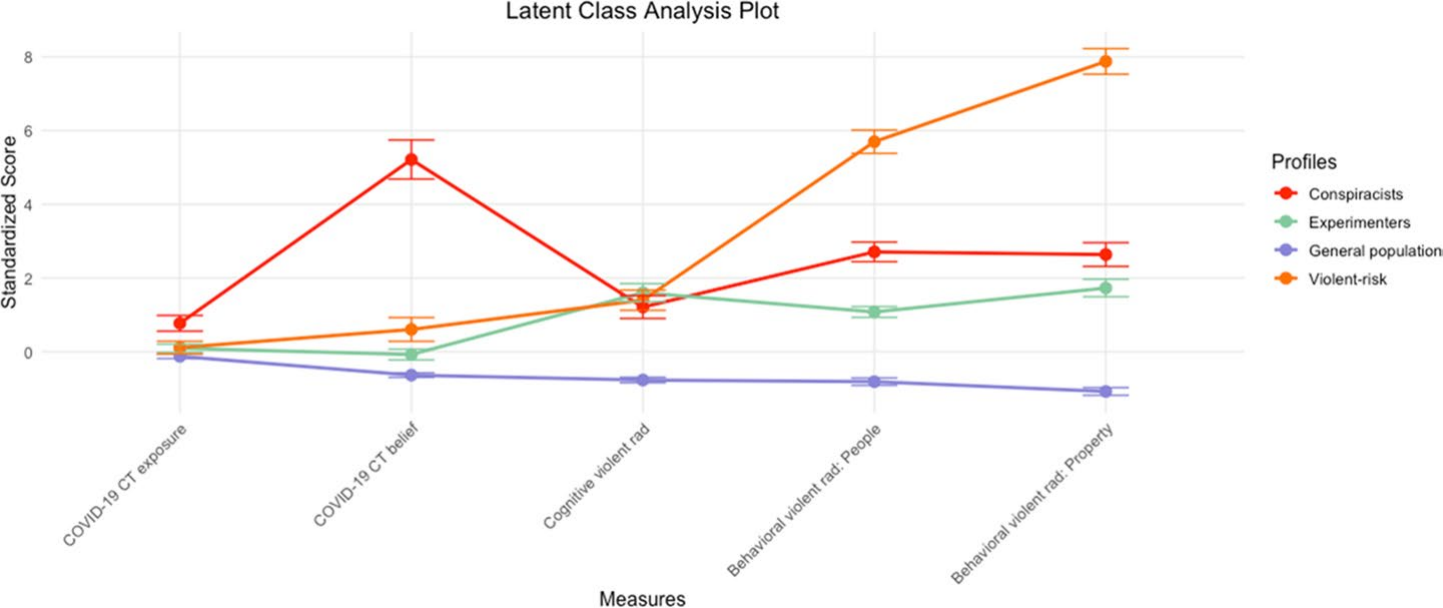
Histogram Latent Factor Scores of Profile Variables of the General Population, Experimenters, Violent-Risk and Conspiracists

In Table 3, standardized latent mean scores along with standard error estimates are presented for profile variables across all subgroups. Figure 1 includes a graphical display of these results and in the Appendix raw means, standard deviations, and frequencies (for behavioral violent radicalization variables) can be found.. The four profiles were named as follow: The general population (*n* = 380, 64%), experimenters (*n* = 121, 20%), violent-risk (*n* = 48, 8%), and conspiracists (*n* = 44, 7%). Most young people belonged to the *general population* subgroup characterized by below average levels COVID-19 CT belief (−0.637) and cognitive (−0.770) and behavioral violent radicalization (people: −0.814; property: −1.080), and modestly lower levels COVID-19 CT exposure (−0.127). The general population subgroup served as a benchmark for comparing the level of profile variables of the other subgroups.

**Table 3:** Comparison of Latent Profile Standardized Means (± SE)

	General Population (*n* = 380)	Experimenters (*n* = 121)	Violent-risk (*n* = 48)	Conspiracists (*n* = 44)
COVID-19 CT exposure	-0.127 (0.060)	0.091 (0.111)*	0.110 (0.172)	0.770 (0.214)***
COVID-19 CT belief	-0.637 (0.061)	-0.078 (0.146)***	0.603 (0.322)***	5.215 (0.530)***
Cognitive violent rad	-0.770 (0.075)	1.602 (0.244)***	1.397 (0.274)***	1.210 (0.309)***
Behavioral violent rad: People	-0.814 (0.100)	1.077 (0.146)***	5.698 (0.316)***	2.708 (0.266)***
Behavioral violent rad: Property	-1.080 (0.104)	1.728 (0.238)***	7.878 (0.347)***	2.636 (0.322)***

*Notes*. Values are STDYX-standardized means (± SE), representing deviations from the overall sample mean in standard deviation units, rad. = radicalization. CT = conspiracy theory, rad. = radicalization

Scores in bold are significantly different from the general population subgroup, after Benjamini–Hochberg correction is applied. The general population subgroup served as a benchmark for comparing the level of profile variables of the other subgroups.

^*^p <.05.; **p <.01.; ***p <.001

The *experimenter* profile (*n* = 121) had nearly average levels of COVID-19 CT exposure (0.091) and belief (−0.078), significantly higher than the general population subgroup. Levels of cognitive violent radicalization (1.602) were also elevated, compared to the general population. This subgroup was called the experimenters due to their slight elevation in behavioral radicalization in the form of violence towards people 1.077) and violence towards property (1.728), all compared to the general population.

The smaller *violent-risk* subgroup (*n* = 48) exhibited near average levels COVID-19 CT exposure (0.110), similar to the general population, but slightly elevated levels of COVID-19 CT belief (0.604). In comparison to the general population subgroup, the violent-risk subgroup also had elevated levels of cognitive violent radicalization (1.397) and behavioral violent radicalization linked to violence towards people (5.698) and property (7.878).

Finally, the smallest subgroup was called the *conspiracists* (*n* = 44) due to above-average levels of COVID-19 CT exposure (0.770) and steeply elevated levels of COVID-19 CT belief (5.215), compared to the general population. Levels of cognitive (1.210) and behavioral violent radicalization, including violence towards people (2.708) and property (2.636), were also elevated compared to the general population.

***Sociodemographic factors***. Table 4 includes an overview of the demographic characteristics per profile, in addition to a comparison between the general population and more at-risk profiles. In terms of age, the general population was, on average, older than the experimenter, violent-risk, and conspiracist profiles. These latter profiles also have significantly higher percentages of males, including 31.2% in the experimenter, 63.1% in the violent-risk, and 35.6% in the conspiracist profile, compared to the general population subgroup (18.9%). No differences were found between the general population and other subgroups on cultural background and educational achievement.

**Table 4:** Demographic factor description of profiles

	General population (*n* = 380)	Experimenters (*n* = 121)	Violent-risk (*n* = 48)	Conspiracists (*n =* 44)
Age (M, se)	21.7 (0.16)	20.5 (0.50)***	19.3 (0.08)***	20.7 (0.41)*
% Male	18.9%	31.2%*	63.1%***	35.6%*
% Native cultural background	93.7%	93.4%	85.4%	81.4%
% Bad/terrible educational achievement (%)	1.3%	1.7%	6.3%	2.2%

*Notes.* Native cultural background = participants who were born in the Netherlands and who had parents who were also born in the Netherlands; Bad/terrible educational achievement = participants who indicated that they did not perform well or performed terribly

*p <.05.; **p <.01.; ***p <.001

***Risk factor presence***. Table 5 presents the means of z-scores and corresponding standard error estimates for each risk factor, as organized per profile. The level of risk factor ranged between below to above average, depending on the variable and profile. Generally, the highest level of risk factors was observed for the violent-risk and conspiracist profiles, compared to the general population. The general population profile consistently had negative z-scores for all risk factors. The experimenter profile had slightly elevated levels for most risk factors, compared to the general population.

**Table 5:** Means and Standard Erros of Z-scored Risk Factors across Latent Profiles (BCH Method)

	General population (*n* = *380*)	Experimenters (*n* = 121)	Violent-risk (*n* = 48)	Conspiracists (*n* = *44)*
*Micro-level risk factors*				
Perceived procedural injustice	-0.102 (0.05)	0.179 (0.10)*	-0.183 (0.14)	0.677 (0.28)**
Perceived police illegitimacy	-0.274 (0.05)	0.261 (0.09)***	0.597 (0.19)***	1.054 (0.20)***
Perceived personal discrimination	-0.236 (0.05)	0.148 (0.09)***	0.589 (0.18)***	1.052 (0.22)***
Aggression	-0.201 (0.05)	0.176 (0.11)**	0.959 (0.22)***	0.137 (0.20)
Impulsiveness	-0.178 (0.06)	0.266 (0.10)***	0.563 (0.17)***	0.053 (0.17)
Changes daily life	-0.081 (0.05)	0.175 (0.13)	0.030 (0.15)	0.179 (0.24)
*Meso-level risk factor*				
Peer delinquency	-0.175 (0.05)	0.071 (0.10)*	1.186 (0.20)***	-0.143 (0.18)

*Notes.* Z-scores in bold are significantly different from the general population, after Benjamini–Hochberg correction is applied

^*^p <.05.; **p <.01.; ***p <.001

**Micro-level.** Compared to the general population, the micro-level risk factors – including *perceived procedural injustice*,* perceived police illegitimacy*,* perceived personal discrimination*,* aggression*, and *impulsiveness* – were all slightly elevated (0.148–0.266) for the experimenter subgroup, compared to the general population. No difference was found between the general population and experimenter subgroup for the risk factor *changes to daily life*.

For the violent-risk profile z-scores were moderately elevated for *perceived police illegitimacy*, *perceived personal discrimination*,* aggression*, and *impulsiveness* (0.563–0.959), compared the general population. No differences were found between the general population and violent-risk subgroup on *perceived procedural justice* and *changes to daily life*.

The conspiracist profile had substantially elevated z-scores for *perceived procedural injustice* (0.677), *police illegitimacy* (1.054), and *personal discrimination* (1.052), compared to the general population. Levels of *impulsiveness* and *changes to daily life* were comparable to the general population.

**Meso-level.** Comparable z-scores were found for the meso-level risk factor of *peer delinquency* between the general population (−0.175) and the conspiracist subgroup (−0.143). The experimenter profile had slightly elevated levels of peer delinquency (0.071), whereas for the violent-risk this was considerably higher (1.186), compared to the general population.

***Power and consistency of findings***. Results from the Monte Carlo Simulation study (see Appendix) demonstrated robust power in detecting the four-profile model, indicative by the significant coefficients above 0.8 for almost all parameters, which means that most simulations correctly identified the sign of the coefficient. For two profiles, power levels for COVID-19 CT exposure (general population, conspiracist) and belief (conspiracist) were low. However, across profiles, the population parameter value for all variables fell within the 95% confidence interval for most replications (92-96% of the cases).

Overall, low bias was detected, as evidenced by low parameter and standard error bias (=5%). The only exception was for COVID-19 CT belief in the general population, and behavioral violent radicalization in the violent-risk profile, with slightly higher standard error bias levels. This suggests that for these profiles there is more variability in the named variables than the model reports.

## Discussion

CTs have taken on a prominent role in public discourse, including the use of violence related to conspiracy belief. The first goal of this study was to investigate profiles of cognitive and behavioral violent radicalization tendencies and COVID-19 conspiracy exposure and belief among a sample of Dutch youth during the COVID-19 pandemic. Relevant demographic factors were assessed to better understand the background of youth in these profiles. The second goal was to test differences between more-and less at-risk profiles in terms of risk factor presence.

### Summary of the Profile Findings

Overall, four different profiles were identified. Most participants (64%) were assigned to the general population subgroup with below-average cognitive and behavioral violent radicalization and COVID-19 conspiracy belief, and average COVID-19 CT exposure. This finding aligns with another person-centered study on youth radicalization, which found that the most prevalent profile exhibited low levels of radical right-wing attitudes (e.g., Schröder et al., 2022).

The other three profiles identified in the current study – named experimenters (20%), violent-risk (8%), and conspiracists (7%) – were distinguished by varying degrees of elevated levels of violent radicalization, compared to the general population, with the conspiracist profile notably defined by its significantly heightened COVID-19 belief and slightly increased exposure.

For the experimenters – named so due to their slight elevation in violent radicalization – cognitive violent radicalization levels were most pronounced. Behavioral violent radicalization was only slightly elevated, aligning with previous research that has shown that sympathizing with the use of violence for one’s social or political goals does not equate to the actual use of violence in this context for most people (Bliesener et al., 2021; McCauley & Moskalenko, 2017).

Similarly elevated levels of cognitive violent radicalization were observed in the violent-risk and conspiracist profiles. However, for the individuals in the violent-risk subgroup, levels of behavioral violent radicalization were most pronounced, including violence towards people and property. Although violence towards property is generally reported more often, most who commit violence against people have also been engaged in violence towards property previously (Carson, 2017; Jugl et al., 2021a).

For most people violent radical attitudes combined with conspiracy belief rarely translate into violent action (Kruglanski et al., 2022; Moskalenko & McCauley, 2021). In the present study, individuals in the conspiracist profile did exhibit some elevation in violent radicalization levels, particularly in cognitive violent radicalization, compared to the general population. This points at the role that conspiracy exposure and belief might play in violent radicalization for some, although the most violently radical young people in this study only had slightly elevated COVID-19 conspiracy belief, and comparable levels of COVID-19 CT exposure to the general population. The conspiracist profile was the only subgroup with above-average COVID-19 CT exposure, compared to the general population. The role that filter bubbles and algorithmic rabbit holes play in exposing people to conspiracy content has previously been highlighted (Schellingerhout et al., 2023). However, most research has focused on standard watch patterns, without considering personal tendencies. It seems important to better understand how self-selected CT exposure might interact with CT belief formation, especially because self-selecting conspiracy related content on YouTube has a stronger effect on signaling conspiracy content than algorithmic pre-selection alone, and one that is harder to reverse (Schellingerhout et al., 2023).

### Summary of Sociodemographic Findings

In line with previous research, individuals in the experimenter, violent-risk and conspiracist profiles were younger and more often male, compared to the general population (Emmelkamp et al., 2020; Wolfowicz et al., 2021). Overall, it has been suggested that associations with sociodemographic factors may be an artifact of actual lived experiences, as both violent radicalization and conspiracy belief have previously been identified as a potential consequence of feelings of powerlessness, or deprivation (Enders et al., 2024; Kunst & Obaidi, 2020).

### Summary of the Risk Factor Findings

Overall, a higher level of risk factors was found in the profiles with higher levels of violent radicalization, compared to the general population. This is in line with previous research that indicates it is the presence and accumulation of risk factors that increases the likelihood for violent radicalization (e.g., Wolfowicz et al., 2021). Accordingly, the higher the level of cognitive and/or behavioral violent radicalization, the more elevated the risk factors.

All risk factors had a higher likelihood to be present in the profiles with elevated levels of COVID-19 exposure and belief and/or cognitive and behavioral violent radicalization. Aggression, impulsiveness, and peer delinquency were particularly pronounced in the violent-risk profile with the highest level of behavioral violent radicalization. This finding corroborates previous research that has demonstrated that individual criminogenic risk factors, such as aggression, have the largest effect on violent radicalization (Wolfowicz et al., 2021). Aggression and peer delinquency are two important risk factors for criminal and antisocial behavior more generally (e.g., Assink et al., 2015a, b), supporting the notion that there is significant overlap in risk factors for behavioral violent radicalization and crime more generally (Lösel et al., 2018, 2020).

No difference was found between the general population and violent-risk profile in terms of perceived procedural injustice. This was somewhat unexpected because procedural justice is an important concept in social, criminological and legal phenomena (e.g., Tyler, 2003). It also seems to be a protective factor against radicalization (Duindam et al., 2025). Perhaps this finding can be explained by the broad way procedural justice was measured in the current study. Our measure focused on the extent to which participants experienced the government as just, whereas much of the previous research on procedural justice has focused on more specific legal authorities (e.g., police, courts). Perhaps people in the violent-risk group – who were more likely to have reportedly engaged in some form of behavioral violent radicalization – had other specific authorities who had been more salient to them than the government. This is also reflected in the finding that police illegitimacy was a risk factor for this group.

Levels of perceived procedural injustice were higher for the conspiracist profile; the essential role of perceived procedural injustice for the development of conspiracy belief has been previously stressed (van Prooijen, 2018). The interrelatedness between conspiracy belief and institutional or governmental distrust, of which perceived procedural injustice can be seen as a component (Murphy et al., 2022), appears quite substantial (Pummerer et al., 2022; van Prooijen et al., 2022a; Prooijen et al., 2022b). Relatedly, perceived police illegitimacy was an important risk factor for the conspiracist profile. A systematic review recently proposed distrust of authorities more generally to be an important antecedent of conspiracy belief (van Mulukom, 2020).

Perceived personal discrimination was another risk factor relevant for the violent-risk, and particularly, the conspiracist profile. This is consistent with previous research that has identified perceived discrimination to have a small effect on youth radicalization and studies that have linked the experience of discrimination and mistreatment to conspiracy belief (Bilewicz, 2022; van Prooijen & Douglas, 2017). Finally, changes in daily life, meaning the extent to which the pandemic stopped individuals from working or extracurricular activities, was similar across profiles.

### Limitations

When interpreting the findings of this study, the following limitations should be considered. First, generalizability of the current findings is limited. COVID-19 conspiracy beliefs were measured, instead of an overarching conspiracy mentality. Although correlations between COVID-19 conspiracy belief and conspiracy mentality are quite considerate (Murphy et al., 2022), a conspiracy mentality is considered to be more stable, less bendable, and overall less influenced by ideological content (Imhoff et al., 2022). Another limitation is that we did not examine the individual association between belief in specific COVID-19 CTs and violent radicalization, even though some prior research has found this relation to be dependent on the nature of the CT (Levinsson et al., 2021).

Second, it is unclear to what extent the same profiles would be found today and outside of the COVID-19 context as the current study was conducted during the COVID-19 pandemic, when CTs were perhaps more salient (van Prooijen & Douglas, 2017). In addition, more female than male participants filled out the survey, in line with other studies that include e-recruitment (Moseson et al., 2020). It has been suggested that the overrepresentation of female participants in online studies might stem from gender differences in how actions in the online environment are valued (Smith, 2008). In general, it is difficult to control who fills in surveys online, although lately some response patterns have been identified (e.g., slightly younger samples, more females, more diverse geographically), which can inform researchers on designing sampling strategies (Moseson et al., 2020).

A third limitation of the current study is its cross-sectional design, like most studies on violent radicalization and conspiracy belief (Hornsey et al., 2022; Wolfowicz et al., 2021). This means that it is unclear to what extent the identified risk factors are precursors or consequences of the conspiracy and violent radicalization constructs.

Finally, it is important to stress that for the current study the aim was to oversample in certain at-risk populations (e.g., during anti-government demonstrations, in telegram groups about conspiracies) to better understand who might be vulnerable for conspiracy-motivated radicalization (Jugl et al., 2021a). As such, we do not assert that different profiles are generalizable or reflective of the actual prevalence of these phenomena in society. Even with the oversampling in at-risk populations, it is important to keep in mind that raw scores of violent radicalization and conspiracy related constructs in the current study were mostly low, including in the more at-risk subgroups (see Appendix). Future research is needed to study the actual presence and magnitude.

### Future Research Directions

Given the stated limitations, future research should explore to what extent current results can be found in larger and more representative samples to allow for more replicable and differentiated conclusions (Lösel, 2018). Additional variables like conspiracy mentality and radical intent can be included to help detect possible at-risk group between the experimenters, who were ‘violence sympathizers’, and the more behaviorally radicalized profiles. In addition, to gain a comprehensive understanding of misinformation’s role in radicalization, a broader measure of misinformation exposure, encompassing both passive (encountering) and active exposure (seeking and sharing content), is needed (e.g., Hassan et al., 2018).

Examining profiles over time could reveal the predictive value of the identified risk factors for conspiracy exposure and belief and violent radicalization. To our knowledge, there are currently no studies on the developmental risk factors of CT belief (Hornsey et al., 2022), and longitudinal studies on radicalization are also scarce (e.g., Nivette et al., 2017). Current findings underscore that the way people navigate and interpret their surroundings holds significance for both CT belief and violent radicalization, which may be linked to their upbringing context (Adam-Troian et al., 2023). Understanding this socio-developmental backdrop could enhance prevention strategies against violent radicalization, with or without conspiracy belief.

It would also be interesting for future research to examine profiles of conspiracy belief and violent radicalization in diverse samples, particularly in at-risk contexts such as people with prior convictions. This is important given the connection between criminality and terrorism (Kupatadze & Argomaniz, 2019). In the current study, non-ideological criminal history was not assessed. Conducting studies across various at-risk contexts is also important because the roles of risk factors shift with radical involvement levels (Becker, 2021); few studies compare risk factors in the general population to those in violent extremist samples (Clemmow et al., 2020).

Finally, an important consideration for research on the topic of conspiracy belief, is that the term ‘conspiracy theory’ is often used pejoratively, while it is an important reality for those who believe in it. By researching this topic, also in relation to violent radicalization, we may have unintentionally contributed to further stigmatization. While it is essential to conduct this kind of research, in the future it is recommended to employ specific strategies (e.g., inviting relevant communities to play a role in research design) to ensure risk for secondary stigmatization is mitigated as much as possible (Millum et al., 2019).

### Prevention Implications

One implication is that varying vulnerability levels of violent radicalization were found in this study with young Dutch people. Most risk factors applied to all profiles that had elevation in COVID-19 belief and/or violent radicalization. The higher the level of these challenges, the higher the risk factor level, in most cases. Thereby, study findings align with the Risk-Need-Responsivity model, universal programs against violent radicalization are sometimes too generic to be effective, tiered interventions are needed that match risk factor levels and target dynamic factors (Andrews & Bonta, 2017; Schröder et al., 2022). Addressing aggression and impulsiveness appear important, while peer delinquency, perceived police illegitimacy and personal discrimination are also intervention targets. Given these factors’ relevance to general crime, anti-radicalization efforts could gain from aligning more closely with anti-crime programs (Jugl et al., 2021b).

For the conspiracist profile, findings highlighted the significance of the risk factors perceived procedural injustice, police illegitimacy and personal discrimination. This emphasizes the importance of ensuring fairness and justice during political decision-making, in order to curb conspiracy belief, especially amid threats to democracy (e.g., pandemic, corruption scandal; van Prooijen, 2011). Whereas previous research has mostly examined the role of micro-level risk factors (including our own work), the few studies into macro-level risk factors suggest a more compassionate picture (Hornsey et al., 2022). Namely, conspiracy belief as response to difficult conditions of adversity and mistreatment, highlighting the overall importance of ensuring the trustworthiness of government and societal institutions, and fair treatment of all groups, to prevent destructive disinformation from gaining traction (Bilewicz, 2022; Hornsey et al., 2022; van Prooijen & Douglas, 2017).

Finally, it is recommended for policy and prevention to counter radical actions rather than radical opinion, which could even create grievances (e.g., Moskalenko & McCauley, 2021). This does not negate the negative impact conspiracy belief and misinformation exposure can have (e.g., Jolley & Douglas, 2014; Van der Linden, 2015). However, for the prevention of actual violence in society, addressing risk factors specific to violently radical behavior is likely more effective. Some countries appear to have developed ineffective, sometimes even counterproductive, security-driven strategies to counter violent radicalization. Moreover, generic programs focusing on youth resiliency might fall short from a public health perspective (Miconi et al., 2021). Therefore, policies are needed to prevent the conditions such as discrimination, adversity, injustice, that might draw young people into violent radicalization and/or conspiracy belief (Stephens & Sieckelinck, 2020).

## Conclusion

This study identified four profiles, named: the general population, experimenter, violent-risk, and conspiracist profile. Micro- and meso-level risk factors were most pronounced in the violent-risk profile, characterized by a higher level of behavioral violent radicalization. The conspiracists exhibited significantly above-average COVID-19 conspiracy belief, and some elevation in violent radicalization as well, and the risk factors perceived police illegitimacy and personal discrimination had the highest impact. The differentiation of these profiles should be considered in focused prevention programs.

## Electronic Supplementary Material

Below is the link to the electronic supplementary material.

Supplementary Material 1

## Data Availability

Data are available upon request by contacting the first author at h.m.duindam@uu.nl.

## Footnotes

1 5-factor model: Cognitive violent radicalization. Behavioral violent radicalization: violence towards people. Behavioral violent radicalization: violence towards property. COVID-19 CT exposure. COVID-19 CT belief.
