# Supplementary material for: Understanding Unrest: Conspiracy Belief and Violent Radicalization Patterns in Young People During the COVID-19 Pandemic in the Netherlands
Source: Eur J Crim Pol Res. 2025 Aug 21;32(2):425–52. doi: 10.1007/s10610-025-09634-z (PMC13279380; doi:10.1007/s10610-025-09634-z)
Supplement: Supplementary file 1 — Supplementary Material 1 [file 10610_2025_9634_MOESM1_ESM.docx]

**Appendix**

| *Results of confirmatory factor analysis 5 factor model* | |  |
| --- | --- | --- |
|  | Fit statistics |  |
| Number of Free Parameters | 94 | |
| Loglikelihood (H0 Value) | 542.036* | |
| Chi-Square Value | 542.036*** | |
| Degrees of Freedom | 465 | |
| RMSEA (Estimate) | 0.022 | |
| RMSEA (90% CI) | 0.016-0.027 | |
| Probability RMSEA ≤ 0.05 | 1.000 | |
| CFI | 0.966 | |
| TLI | 0.963 | |
| SRMR | 0.066 | |

*Latent Profile Standardized Means (± SE) for 1-. 2-, 3-, 5-profiles model solution*

|  | CCT Exposure | CCT belief | Rad attitudes | Violent rad. | Rad. vandalism |  |
| --- | --- | --- | --- | --- | --- | --- |
| Profiles | *M (se)* | *M (se)* | *M (se)* | *M (se)* | *M (se)* | *N* |
| 1 | 0.002 (0.04) | 0.001 (0.04) | 0.021 (0.04) | 0.164 (0.04) | 0.176 (0.04) | 593 |
|  |  |  |  |  |  |  |
| 2 | -0.108 (0.05) | -0.315 (0.05) | -0.319 (0.07) | -0.382 (0.05) | -0.364 (0.06) | 480 |
|  | 0.470 (0.13) | 1.352 (0.20) | 1.494 (0.15) | 3.087 (0.24) | 3.125 (0.23) | 113 |
|  |  |  |  |  |  |  |
| 3 | -0.108 (0.05) | -0.359 (0.04) | -0.683 (0.078) | -0.684 (0.07) | -0.956 (0.09) | 413 |
|  | 0.307 (0.12) | 0.932 (0.21) | 1.764 (0.16) | 1.617 (0.16) | 2.230 (0.17) | 130 |
|  | 0.106 (0.16) | 0.542 (0.23) | 1.373 (0.27) | 5.390 (0.25) | 7.798 (0.30) | 50 |
|  |  |  |  |  |  |  |
| 5 | -0.135 (0.06) | -0.764 (0.08) | -0.783 (0.08) | -0.869 (0.11) | -1.109 (0.10) | 379 |
|  | 0.119 (0.11) | -0.076 (0.17) | 1.611 (0.25) | 1.153 (0.14) | 1.755 (0.24) | 123 |
|  | 0.757 (0.21) | 6.235 (0.52) | 1.183 (0.32) | 2.789 (0.27) | 2.620 (0.34) | 42 |
|  | -0.219 (0.17) | -0.501 (0.18) | 1.049 (0.33) | 5.391 (0.30) | 7.608 (0.35) | 37 |
|  | 1.150 (0.34) | 4.587 (0.78) | 2.532 (0.41) | 7.925 (0.58) | 9.261 (0.71) | 12 |
|  |  |  |  |  |  |  |

*Raw Means (± SD) for 4-profile solution*

|  | CCT Exposure (range: 1-4) | CCT belief (range: 1-5) | Rad attitudes (range: 1-5) | Violent rad. (range: 0-1) | Rad. Vandalism (range: 0-1) |
| --- | --- | --- | --- | --- | --- |
| General Population | 1.76 (0.61) | 1.11 (0.25) | 1.33 (0.33) | 0.00 (0.01) | 0.00 (0.00) |
| Experimenters | 1.91 (0.67) | 1.32 (0.41) | 2.54 (0.45) | 0.00 (0.02) | 0.00 (0.00) |
| Violent-risk | 1.88 (0.74) | 1.55 (0.79) | 2.35 (0.97) | 0.17 (0.27) | 0.40 (0.27) |
| Conspiracists | 2.27 (0.87) | 3.39 (0.73) | 2.25 (0.78) | 0.01 (0.03) | 0.01 (0.04) |

*Items of violent radicalization towards people, number of participants (%) who engaged in this behavior at least once*

| *Have you ever… because of their political or religious beliefs* | *Fought with someone* | *Threatened anyone on the internet* | *Threatened someone in the streets* | *Hit someone* | *Thrown stones at the police* |
| --- | --- | --- | --- | --- | --- |
| General Population | 0% | 0% | 0.3% | 0% | 0% |
| Experimenters | 0% | 0% | 0% | 0% | 0.8% |
| Violent-risk | 14.6% | 33.3% | 10.4% | 12.5% | 14.6% |
| Conspiracists | 0% | 2.3% | 0% | 0% | 0% |

*Items of violent radicalization towards property, number of participants (%) who engaged in this behavior at least once*

| *Have you ever… because of their political or religious beliefs* | Written a political message of political graffiti on a wall | Vandalised anything in the street or at a station | Damaged someone’s property | Set something on fire |
| --- | --- | --- | --- | --- |
| General Population | 0% | 0% | 0% | 0% |
| Experimenters | 0% | 0% | 0% | 0% |
| Violent-risk | 25% | 39.6% | 60.4% | 35.4% |
| Conspiracists | 2.3% | 0% | 0% | 0% |

*Monte Carlo Simulation Study Results*

|  | Variable | | Population | Average | Parameter bias | Std. Dev. | S.E. Average | S.E. Average bias | M.S.E. | 95% Cover. | % Sign. Coef |
| --- | --- | --- | --- | --- | --- | --- | --- | --- | --- | --- | --- |
| **General Population** |  |  |  |  |  |  |  |  |  |  |  |
|  | Means | CCT Exposure | 0.065 | 0.0626 | 3.692 | 0.0841 | 0.0842 | 0.1189 | 0.0071 | 0.935 | 0.120 |
|  |  | CCT Belief | 0.245 | 0.2434 | 0.6530 | 0.0638 | 0.0577 | -9.5611 | 0.0041 | 0.919 | 0.977 |
|  |  | Cog. Rad. | 0.564 | 0.5673 | -0.5851 | 0.0597 | 0.0575 | -3.6850 | 0.0036 | 0.939 | 1.000 |
|  |  | Beh. Rad. Vio | 1.457 | 1.4576 | -0.0411 | 0.0367 | 0.0364 | -0.8174 | 0.0013 | 0.944 | 1.000 |
|  |  | Beh. Rad. Van | 1.572 | 1.5719 | 0.0063 | 0.0292 | 0.0286 | -2.0548 | 0.0009 | 0.939 | 1.000 |
|  |  |  |  |  |  |  |  |  |  |  |  |
|  | Variances | CCT Exposure | 0.349 | 0.3463 | 0.7736 | 0.0202 | 0.0200 | -0.9901 | 0.0004 | 0.936 | 1.000 |
|  |  | CCT Belief | 0.164 | 0.1632 | 0.4878 | 0.0099 | 0.0095 | -4.0404 | 0.0001 | 0.930 | 1.000 |
|  |  | Cog. Rad. | 0.163 | 0.1616 | 0.8589 | 0.0099 | 0.0099 | 0 | 0.0001 | 0.941 | 1.000 |
|  |  | Beh. Rad. Vio | 0.065 | 0.0647 | 0.4615 | 0.0039 | 0.0039 | 0 | 0.0000 | 0.950 | 1.000 |
|  |  | Beh. Rad. Van | 0.040 | 0.0398 | 0.5 | 0.0025 | 0.0025 | 0 | 0.0000 | 0.936 | 1.000 |
| **Experimenters** |  |  |  |  |  |  |  |  |  |  |  |
|  | Means | CCT Exposure | 0.455 | 0.4573 | 0.8 | 0.0925 | 0.0886 | -4.2162 | 0.0085 | 0.929 | 0.996 |
|  |  | CCT Belief | 2.114 | 2.114 | 0.3488 | 0.0638 | 0.0628 | -1.5674 | 0.0041 | 0.939 | 1.000 |
|  |  | Cog. Rad. | 0.489 | 0.489 | 0.3680 | 0.0637 | 0.0608 | -4.5526 | 0.0041 | 0.947 | 1.000 |
|  |  | Beh. Rad. Vio | 0.692 | 0.692 | -0.0480 | 0.0398 | 0.0385 | -3.2663 | 0.0016 | 0.934 | 1.000 |
|  |  | Beh. Rad. Van | 0.526 | 0.526 | 0.0926 | 0.0312 | 0.0303 | -2.8846 | 0.0010 | 0.937 | 1.000 |
|  |  |  |  |  |  |  |  |  |  |  |  |
|  | Variances | CCT Exposure | 0.349 | 0.3463 | 0.77365 | 0.0202 | 0.0200 | -0.9901 | 0.0004 | 0.936 | 1.000 |
|  |  | CCT Belief | 0.164 | 0.1632 | 0.4878 | 0.0099 | 0.0095 | -4.0404 | 0.0001 | 0.930 | 1.000 |
|  |  | Cog. Rad. | 0.163 | 0.1616 | 0.8588 | 0.0099 | 0.0099 | 0 | 0.0001 | 0.941 | 1.000 |
|  |  | Beh. Rad. Vio | 0.065 | 0.0647 | 0.4615 | 0.0039 | 0.0039 | 0 | 0.0000 | 0.950 | 1.000 |
|  |  | Beh. Rad. Van | 0.040 | 0.0398 | 0.5 | 0.0025 | 0.0025 | 0 | 0.0000 | 0.936 | 1.000 |
| **Violent** |  |  |  |  |  |  |  |  |  |  |  |
|  | Means | CCT Exposure | -0.075 | -0.0744 | 0.8 | 0.0299 | 0.0305 | 2.0067 | 0.0009 | 0.952 | 0.0684 |
|  |  | CCT Belief | -0.258 | -0.2571 | 0.34883 | 0.0220 | 0.0209 | -5 | 0.0005 | 0.932 | 1.000 |
|  |  | Cog. Rad. | -0.311 | -0.3114 | -0.12861 | 0.0209 | 0.0212 | 1.4354 | 0.0004 | 0.953 | 1.000 |
|  |  | Beh. Rad. Vio | -0.208 | -0.2081 | -0.04808 | 0.0143 | 0.0133 | -6.9930 | 0.0002 | 0.938 | 1.000 |
|  |  | Beh. Rad. Van | -0.216 | -0.2158 | 0.09259 | 0.0109 | 0.0106 | -2.7522 | 0.0001 | 0.943 | 1.000 |
|  |  |  |  |  |  |  |  |  |  |  |  |
|  | Variances | CCT Exposure | 0.349 | 0.3463 | 0.7736 | 0.0202 | 0.0200 | -0.9900 | 0.0004 | 0.936 | 1.000 |
|  |  | CCT Belief | 0.164 | 0.1632 | 0.4878 | 0.0099 | 0.0095 | -4.0404 | 0.0001 | 0.930 | 1.000 |
|  |  | Cog. Rad. | 0.163 | 0.1616 | 0.8589 | 0.0099 | 0.0099 | 0 | 0.0001 | 0.941 | 1.000 |
|  |  | Beh. Rad. Vio | 0.065 | 0.0647 | 0.4615 | 0.0039 | 0.0039 | 0 | 0.0000 | 0.950 | 1.000 |
|  |  | Beh. Rad. Van | 0.040 | 0.0398 | 0.500 | 0.0025 | 0.0025 | 0 | 0.0000 | 0.936 | 1.000 |
| **Conspiracists** |  |  |  |  |  |  |  |  |  |  |  |
|  | Means | CCT Exposure | 0.054 | 0.0522 | 3.3333 | 0.0551 | 0.0554 | 0.5445 | 0.0030 | 0.943 | 0.152 |
|  |  | CCT Belief | -0.031 | -0.0303 | 2.2581 | 0.0376 | 0.0383 | 1.8617 | 0.0014 | 0.948 | 0.114 |
|  |  | Cog. Rad. | 0.647 | 0.6497 | -0.4173 | 0.0397 | 0.0392 | -1.2594 | 0.0016 | 0.942 | 1.000 |
|  |  | Beh. Rad. Vio | 0.275 | 0.2743 | 0.2545 | 0.0244 | 0.0246 | 0.8197 | 0.0006 | 0.942 | 1.000 |
|  |  | Beh. Rad. Van | 0.345 | 0.3454 | -0.1159 | 0.0197 | 0.0197 | 0 | 0.0004 | 0.958 | 1.000 |
|  |  |  |  |  |  |  |  |  |  |  |  |
|  | Variances | CCT Exposure | 0.349 | 0.3463 | 0.7736 | 0.0202 | 0.0200 | -0.9901 | 0.0004 | 0.936 | 1.000 |
|  |  | CCT Belief | 0.164 | 0.1632 | 0.4878 | 0.0099 | 0.0095 | -4.0404 | 0.0001 | 0.930 | 1.000 |
|  |  | Cog. Rad. | 0.163 | 0.1616 | 0.8589 | 0.0099 | 0.0099 | 0 | 0.0001 | 0.941 | 1.000 |
|  |  | Beh. Rad. Vio | 0.065 | 0.0647 | 0.4615 | 0.0039 | 0.0039 | 0 | 0.0000 | 0.950 | 1.000 |
|  |  | Beh. Rad. Van | 0.040 | 0.0398 | 0.5 | 0.0025 | 0.0025 | 0 | 0.0000 | 0.936 | 1.000 |
